# A rapid scoping review of fear of infertility in Africa

**DOI:** 10.1186/s12978-020-00973-0

**Published:** 2020-09-14

**Authors:** Jacky Boivin, Judith Carrier, Joseph Mumba Zulu, Deborah Edwards

**Affiliations:** 1 School of Psychology. College of Biomedical and Life Sciences, Cardiff University, 70 Park Place, CF10 3AT Cardiff, Wales, UK; 2 Wales Centre for Evidence Based Care, School of Health Care Sciences, Cardiff University, Cardiff, Wales UK; 3 School of Public Health, University of Zambia, Lusaka, Zambia

**Keywords:** Infertility, Health education, Family planning service provision, Counselling, Qualitative research

## Abstract

**Background:**

Fear of infertility (FOI) is often reported in studies about reproductive health but this literature not yet mapped. The aim of this rapid scoping review of qualitative studies was to describe the nature of FOI in Africa.

**Methods:**

Eligibility criteria were qualitative data from Africa reporting views of women and men of any age. MEDLINE and CINAHL databases were searched for English language citations to February 2019 using keywords related to fear, infertility and Africa. Two independent reviewers screened texts for inclusion.

**Results:**

Of 248 citations identified, 38 qualitative and six review papers were included. FOI was reported in diverse groups (e.g., men, women, fertile, infertile, married, unmarried, teachers, religious leaders). Two types of fears were identified: (1) fear of triggering infertility due to specific reproductive choices and (2) fear of the dire future consequences of infertility. Choices were perceived to affect fertility via internal accumulation and blockage (e.g., of menstrual blood), structural damage (e.g., burnt eggs), internal movement of contraceptive material, deliberate toxicity preventing population growth and behavioral effects impeding sexual activity. Diverse feared consequences of infertility were reported (e.g., polygamy, economic hardships). Fears were reported to affect reproductive behaviour (e.g., stopping contraception), help-seeking and social behaviour.

**Conclusion:**

FOI is a phenomenon that should be studied in its own right. Fears could originate from genuine threats, incorrect knowledge, distortions of truths, or dissemination of false information. Rigorous studies are needed to better understand FOI and integrate it in health education, client counselling and family planning service provision.

## Plain English summary

Parenthood is one of the most desired and valued goals of adulthood. Due to this importance some past studies in Africa have reported that people fear having fertility problems, known as fear of infertility. Not much is known about who reports fear of infertility, what the fear is about or how it affects health behaviours. To learn more about it we searched databases and identified studies in Africa providing descriptions of fear of infertility from men and women. In total 44 published records were examined in detail and summarised. The results showed that fear of infertility was reported by many groups (e.g., married, unmarried, fertile or not, doctors, teachers, religious leaders, men, women). Fear presented itself in two ways. First, people feared triggering infertility because of the choices they made for example, using a particular type of family planning or having certain vaccinations. Many reasons were given for why choices might affect fertility (e.g., damaging insides, accumulation of blood). Second, people feared the dire consequence of being infertile for example, being excluded from their communities or divorced by husbands or wives. Fears were reported to affect health behaviour, for example, not using family planning properly or doing treatments that could cause more harm to fertility. The review concluded that fear of infertility was a real phenomenon that should be studied in its own right, that education should be provided to address fears and that more research should be conducted on why it existed.

## Background

Fear is an expectation of negative outcomes that is constructed from a complex interplay of physical, psychological, social and cultural relations [1]. One fear that is reported as impacting reproductive choices globally is fear of infertility (FOI), a fear associated with a future inability to achieve pregnancy or father a child [2–4]. Fear of Infertility often presents in the context of decision-making about family planning or other health choices affecting reproductive organs (e.g., cervical screening) [3–5]. Fear of infertility is critical to understand and address because it is often unfounded [2], persists from adolescence to adulthood and can have adverse effects on health [3, 6–8]. Fear of infertility is strongest where childlessness is most stigmatised, in rural areas of lowest functional and health literacy [4] and where childlessness is associated with severe consequences especially for women [9, 10]. The research referring to FOI has not yet been mapped.

A scoping rapid review approach was chosen and performed according to established methods [11]. A rapid review provides high-quality evidence and knowledge synthesis using a stream-lined review process (e.g., searching fewer databases, restricted search timeframe, omitting critical appraisal) [12]. We focused on synthesis of qualitative studies as the design most likely to generate data that would describe FOI and its nature. This approach was selected to achieve the mapping process within the project timeframe of 3 months. We focused on Africa because this review was part of a programme of activities relating to infertility in Zambia prioritised by the Ministry of Health to support integration of fertility care in reproductive health policy and services. The programme of research was developed via face-to-face discussions with academics, healthcare professionals and policymakers who helped identify and prioritise the infertility research strands, outcomes and dissemination strategies. In this programme we also conducted drawing workshops with young married and unmarried women.

## Review aim

The aim of this review of qualitative studies was to map and describe main concepts related to FOI from the perspectives of men and women in African countries.

## Main text

### Methods

#### Inclusion criteria

This review considered studies that: (1) referred to or explored FOI and what the fears concerned or affected; (2) provided views of women and men of any age from African countries, and; (3) had a qualitative design including mixed methods designs where qualitative data could be extracted separately. Ethical review was not required. The project proposal and all study materials will be available through Open Science Framework (link to be inserted after review). Studies were excluded if they did not explore either ‘fear’ of triggering infertility or ‘fear’ of consequence of infertility. Non-African countries were excluded as were quantitative papers.

#### Search strategy

MEDLINE (on the OVID platform) and CINAHL (on the EMBSCO platform) were searched for English language citations for published material from database inception to February 2019 using keywords fear AND infertil* OR childless* OR infecundity OR subfecundity AND Africa* OR list of names of all African countries. A separate search was conducted using the term contraceptive OR family planning AND terms for infertility (see Additional file 1 Search History). The reference list of all included studies was screened for additional studies. Medline and CINAHL were chosen as they are the main recommended databases for sourcing relevant studies when conducting a rapid review.

#### Study screening and selection

Citations were loaded into Endnote and duplicates removed. Two independent reviewers screened titles, abstracts and full texts of potentially relevant studies using a pre-piloted screening tool designed for the study. Any disagreements were resolved through discussion.

#### Data extraction

Data extracted were participant demographic characteristics (e.g., region and country, participants, age), study aims, recruitment, design, questions that elicited FOI data, findings related to FOI, nature of specific fears and reported consequences of FOI. Extraction was conducted by one reviewer and checked by a second. Only data with relevance to FOI were extracted.

#### Assessment of methodological quality

An assessment of methodological quality was not conducted which is consistent with accepted scoping review methods [11].

#### Presentation of data

Data were extracted into tables and a narrative summary provided. For the demographic characteristics data were tabulated using the following headings: region and country, participants and recruitment, methods of data collection, age, ethnicity and religion. A narrative summary accompanies the tabulated results describing how the results related to the review objectives and question. The Preferred Reporting Items for Systematic Reviews and Meta-Analyses Extension for Scoping Reviews (PRISMA_SCr) checklist has been followed for the reporting of this review (see Additional File 2 PRISMA_SCr).

## Results

### Study inclusion

**Fig. 1:**
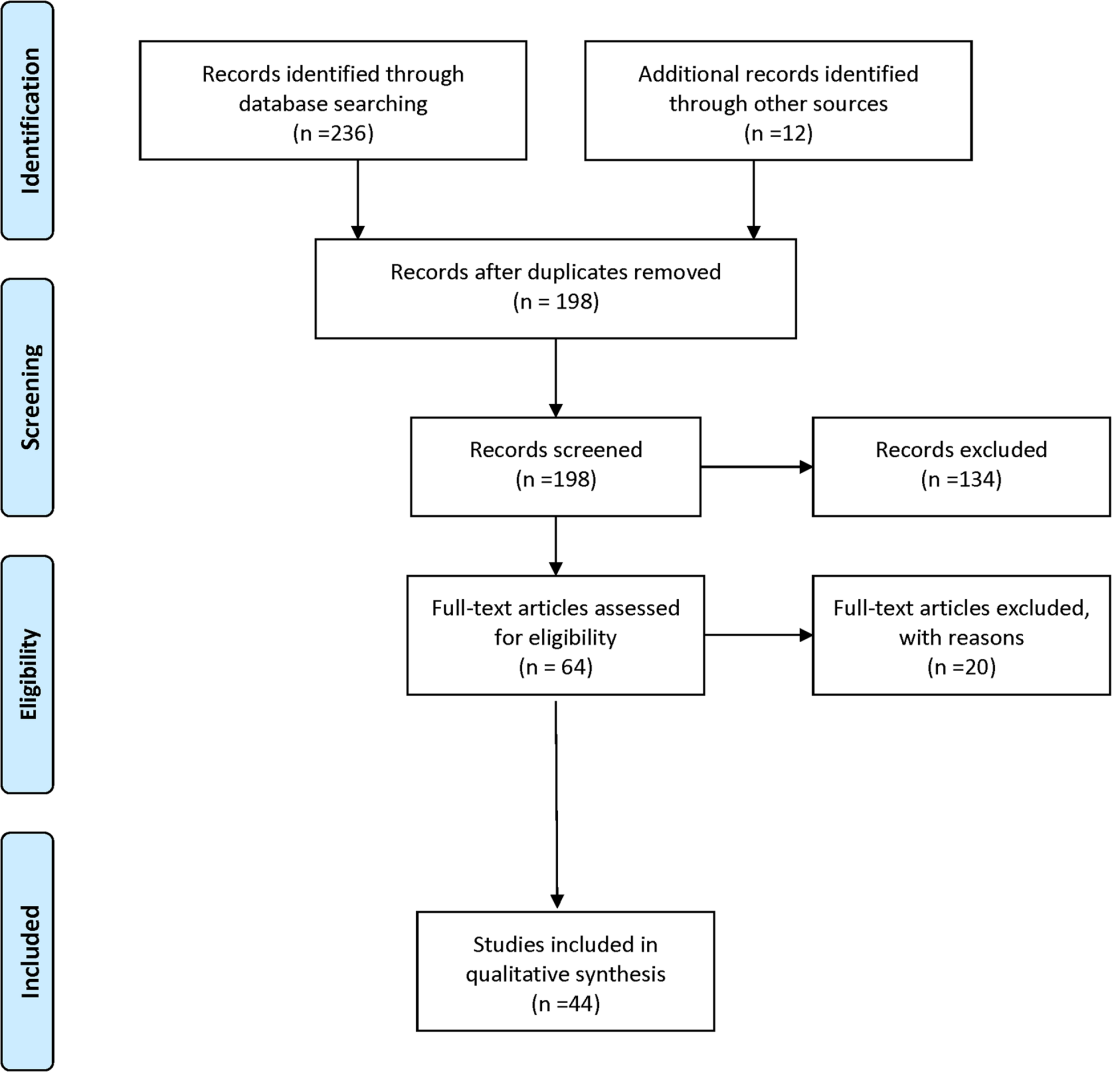
Study flow chart. Flow chart from: Moher D, Liberati A, Tetzlaff J, Altman DG, The PRISMA Group (2009). Preferred Reporting Items for Systematic Reviews and Meta-Analyses: The PRISMA Statement. PLoS Med 6(7): e1000097. doi:10.1371/journal.pmed1000097

Figure 1 shows the PRISMA flow diagram for study selection process. Of 248 citations identified, 64 full-text studies were assessed for eligibility and a total of six review papers and 38 qualitative and mixed methods papers (representing 37 studies) were included. Twenty full-text studies did not meet the inclusion criteria (listed in Additional File 3 Excluded studies).

## Characteristics of the included studies

### Phenomena of interest

Review of included studies showed two types of fears were reported: (1) fear of triggering future infertility due to specific reproductive or health choices (hereafter ‘triggering infertility’ studies), and; (2) fear of the dire consequences of infertility should one prove unable to demonstrate fertility (hereafter ‘infertility consequences’ studies).

The characteristics of individual studies are reported in Tables 1 and 2 (respectively) and of review studies (which could concern both types) in Table 3.

**Table 1:** Characteristics of included studies related to ‘triggering infertility’

Author/s Region, Country	Methods of data collection Participants and Recruitment	Focus of study Age (years)
**a) Qualitative descriptive part of a mixed methods study**		
1.Dalaba et al. 2016 [13] Kassena-Nankana, Ghana	Focus groups (*n* = 16) with men and women (*n* = ns) from community-based health planning & services Interviews with community Chiefs and Elders (*n* = 8)	Hormonal contraceptives > 35 or < 35
2. Morse et al. 2012 [14] Kampala, Uganda	Focus groups (*n* = 10) with pregnant women (*n* = 46) presenting for prenatal care at local hospital	General contraception and FP < 20 (*n* = 7) / 21–25 (*n* = 19) 26–30 (*n* = 11) / > 30 (*n* = 9)
3. Capurchande et al. 2016 [15] Ndlavela & Boane, Mozambique	Focus groups (*n* = 4); interviews (*n* = 16), informal conservations (*n* = 4); Observations with adolescents and young adults (F: *n* = 23, M: *n* = 19) selected from respondents to wider community survey	General contraceptive methods Range 15–24
4. Gebremariam and Addissie 2014 [16] Adigrat town & Tigray, Ethiopia	Focus groups (*n* = 5) with married men and women (*n* = ns) selected from wider community survey and interviews with FP service providers (*n* = 6) selected from HCPs in local health centres	LAPCM Range 15–49
5. Koster 2010 [6] Yoruba, Nigeria	Interviews with women with fertility problems (*n* = 223) who had completed a community survey or those who had participated in the development of the survey	Abortion Range 15–49
**b) Qualitative descriptive part of a randomised control trial**		
6. Chituka et al. 2019 [17] Lilongwe (Malawi); Cape Town, Durban, Johannesburg (South Africa); Kampala (Uganda); Harare (Zimbabwe)	Single Interviews (*n* = 34), serial interviews at 3 months, 6 months and product end (*n* = 80) and focus groups (*n* = 100 participants) with healthy sexually active HIV-negative women (*n* = 214)	Vaginal ring Mean 26.4 Range 14–42
**c) Qualitative descriptive**		
7. Castle 2003 [18] Barnako & Sikasso, Mali	Interviews with adolescent (M: n = 10, F *n* = 10) from peer education programs, adolescents (M: *n* = 10, F: *n* = 10) from community, peer educators (M: n = 10, F *n* = 10); HCPs (M: *n* = 4 F *n* = 4)	Hormonal contraceptives Range 15–19
8. Cover et al. 2017 [19] Gulu District, Uganda	Interviews with adolescent women (*n* = 46) from an outreach clinic and youth centre	Contraceptive self injection Range 15–19
9. Hyttel et al. 2012 [20] Mbarara & Kampala, Uganda	Interviews (F: *n* = 28; M: *n* = 18) recruited while waiting for health services, while attending NGO activities, identified by Reproductive Health Uganda peer educators or randomly from their villages Focus groups (*n* = 3) with FP service providers (*n* = 17) working across public and private sectors, policymakers (*n* = 15) selected from organizations and snowball sampling	Injectable hormonal contraceptives F; 18–29 (*n* = 9) / F: 30–45 (*n* = 19) M: 18–29 (*n* = 9) / M: 30–60 (*n* = 9)
10. Krugu et al. 2017 [21] Bolgatanga, Ghana	Interviews with young women who have experienced pregnancy (*n* = 20) recruited through advertisements in public buildings, including schools and health or by nurses at local health centres	General contraception and FP Range 14–19
11. Muanda et al. 2016 [22] Kinshasa, DRC	Focus groups (*n* = 10) with women and their husbands who had at least two children (*n* = ns) recruited from private and public health centres	General contraception and FP 20–34 (married); 15–19 (unmarried)
12. Adongo et al. 2014 [23] SBAB & KEEA, Ghana	Focus groups (*n* = 21) with men (*n* = ns) and Women (*n* = ns) married with children from the community Interviews with CH officers; HC volunteers and HCM from the community	General contraceptive methods Not reported
13. Ndwamato and Ogunbanjo 2009 [24] Limpopo Province, South Africa	Focus groups (*n* = 5) with multiparous women (*n* = ns) seen at a local hospital	General contraception and FP Not reported
14. Otoide et al. 2001 [7] Benin City, Nigeria	Focus groups (*n* = 20) with women (*n* = 149) who were sexually active & those who had not initiated sexual activity who were selected on the basis of their current vocation or pursuit within Benin City	Abortion Range 15–24
15. Schuster 2005 [8] Anglophone, Cameroon Grassfields	Interviews and participant observation with women who had come to the hospital for treatment of complications of unsafe abortion or who had an induced abortion in their history (*n* = 58) identified through medical records and women who had had an abortion and had not been hospitalised identified through a snowball sample (*n* = 7). Interviews with key informants (*n* = ns)	Abortion Not reported
16. Lunsford et al. 2017 [5] Nairobi & Nyanza, Kenya	Focus groups (*n* = 10) with women (*n* = 60) and their partners (*n* = 40) who had received cervical cancer screening (*n* = 60) and those who did not (*n* = 40) recruited from health care and community forums	Cervical screening Range 25–49
17. Remes et al. 2012 [25] Mwanza Region & Misungwi, Tanzania	Focus groups (*n* = 12) and interviews with female students (*n* = 54) from local schools, teachers (*n* = 19); Parents (*n* = 59), health workers (*n* = 9), religious leaders (*n* = 9)	Vaccination Students: 11–17
**d) Ethnographic studies**		
18. Ochako et al. 2015 [26] Kismu, Mombasa &, Thika, Kenya	Interviews with sexually active women both users (*n* = 20) and non-users of contraceptives (*n* = 11) purposively selected from the community	General contraception and FP 16–19 (*n* = 13) / 20–24 (*n* = 11)
19. Klinger and Asgary 2017 [27] Anivorano Nord, Ambondromifehy, Marotaolana, and Beanemalao; Madagascar	Focus groups (*n* = 7) with adolescents (F: *n* = 23 / M: *n* = 20) residing in or attending local schools Interviews with those in each of the four villages who were involved with providing medical care or education to the youth in the village (Physician F: *n* = 1, Midwives F: *n* = 2, CH Workers *n* = 2) & Aid workers (*n* = 2)	General contraceptive methods Range 15–19
20. Chebet et al. 2015 [28] Morogoro Region, Tanzania	Interviews with postpartum women (*n* = 34), their partners (*n* = 23), community leaders (*n* = 12); CH leaders (*n* = 19); Facility health providers (*n* = 12) recruited from local communities	General contraceptive methods F: Mean 28.56 / F: Range 18–43
21. Sedlander et al. 2018 [29] Kilifi County, Kenya	Focus groups (*n* = 32) with men, women, adolescent boys and girls (*n* = 153) and interviews with village chiefs and elders, pastors, teachers, health care workers (*n* = 10) from the community.	General contraception and FP Mean 26.2 / Range 13–65

Key: *CH* community health; *DRC* Democratic Republic of Congo; *F* female; *FP* family planning; *HCM* health care managers; *HCP* Health care providers; *KEEA* Komenda-Edina-Eguafo-Abrem; *LAPCM* Long acting and permanent contraceptive methods; *M* Male; *SBAB* Sefwi Bibiani-Ahwiaso Bekwai. Reference citation follows author name in square brackets

**Table 2:** Characteristics of included studies related to “infertility consequences”

Author/s Region, Country	Methods of data collection Participants and Recruitment	Age (years)
**a) Qualitative descriptive part of a mixed methods study**		
1.Dhont et al. 2011 [30] Kigali, Rwanda	Focus group discussions (*n* = 5) with couples (F: *n* = 21 / M: *n* = 20) with infertility problems being offered investigations at an Infertility clinic	F: Mean 28.5 / Range 27–33 M: Mean 34.5 / Range 30–40
2.Donkor et al. 2017 [31] Accra, Ghana	Interviews with women (*n* = 14) receiving treatment for infertility problems at a local hospital	Range 27–42
3.Dyer et al. 2002 [32] South Africa	Interviews with women (*n* = 30) receiving treatment for infertility problems at an infertility clinic	Mean 31.5 / Range 21–41
4.Hess et al. 2018 [33] Koutiala, Mali	Interviews with infertile women (*n* = 26) attending a hospital infertility clinic	Mean 17–44
5.Dierickx et al. 2018 [10] West Coast region, The Gambia	Interviews with infertile women (*n* = 33) from the local community	> 18
6.Hollos and Larsen 2008 [34] Moshi, Tanzania	Interviews with infertile (*n* = 25) and fertile women (*n* = 25) from the local community	Range 20–44
**b) Qualitative descriptive studies**		
7. Fledderjohann 2012 [35] Accra, Ghana	Interviews with women (*n* = 107) seeking treatment in gynaecological and obstetric clinics	Mean 33 Range 21–48
8. Mabasa 2005 [36] South Africa	Interviews with infertile couples (*n* = 10) and infertile women (*n* = 9) selected through researchers’ networks and snowball sampling	Mean 36.9 Range 25–48
9. Runganga et al. 2001 [37] Harare, Zimbabwe	Focus group discussions(*n* = 9) and interviews with women (*n* = 8) and men (*n* = 2) attending a fertility clinic for reproductive problems	Mean 30 Range 21–40
10. Tabong and Adongo 2013a/b [38, 39] Upper West Region, Ghana	Focus groups (*n* = ns) and interviews with childless couples (*n* = 15) selected by CH volunteers and snowball sampling and gynaecologists (*n* = 2); Islamic scholar (*n* = 1); Christian leader (*n* = 1); traditional medical practitioners (*n* = 2); manager of NHIS (*n* = 1); manager PIC (*n* = 1)	F: Range 28–52 M: Range 35–63
11. Naab and Kwashie 2018 [40] Ghana	Interviews with married men (*n* = 12) receiving treatment for infertility at a local hospital	> 25 years Range 29–41
**c) Qualitative phenomenological studies**		
12. Kamau 2012 [41] Nairobi Province, Kenya	Interviews with infertile women (*n* = 10) attending local churches	Mean 40.4 Range 29–54
**d) Anthropological studies**		
13. Gerrits 1997 [42] Montepuez, Mozambique	Interviews with infertile (*n* = 34) and fertile women (*n* = 10) from the local community and traditional healers (*n* = 3); midwives (*n* = 3); physicians (*n* = 2); nurses (*n* = 3)	Range 19–50
14. Feldman-Savelsberg 1994 [43] Bangangte, Cameroonian Grassfields	Narrative with infertile women (no further details provided)	not reported
**e) Ethnographic studies**		
15. Upton and Dolan 2011 [44] Northern Botswana	Ethnographic narratives with men (*n* = 20) and women (*n* = 31) who were married, unmarried, fertile and those identifying to have struggled with fertility problems selected from local community	not reported
16. Parrott 2014 [45] Karonga District, Malawi	Life history interviews with men who had experienced childless marriages (*n* = 55) selected from a wider community survey	not reported

Key: *CH* Community health; *F* Females; *M* Males. *NHIS* National Health Insurance Scheme; *PIC* Private Insurance company. Reference citation follows author name in square brackets

**Table 3:** Characteristics of included review articles

Author	Type of review	Country	Focus
1.Polis et al. 2018 [46]	Scoping review	Africa (11%)	Women’s responses to contraceptive-induced menstrual bleeding changes
2.Ackerson and Zielinski 2017 [47]	Narrative review	Sub-Saharan Africa	Factors that inhibit or promote family planning and contraceptive use
3.Dyer and Patel 2012 [48]	Systematic evaluation	Developing countries Africa (*n* = 13)	Out-of-pocket payment for infertility care
4.Daniele et al. 2017 [3]	Systematic review	Low- and middle-income countries Including Africa	Provider and lay perspectives on intra-uterine contraception
5.Williamson et al. 2009 [4]	Systematic review	Developing countries Sub-Sahara Africa (*n* = 6)	Limits to modern contraceptive use identified by young women
6.van Balen and Bos 2009 [9]	Literature review with adapted IPA	Poor resource areas Sub-Sahara Africa (*n* = 19)	Social and cultural effects of being childless

Key: *IPA* interpretative phenomenological analysis. Reference citation follows author name in square brackets

For ‘triggering infertility’, 21 studies and 4 review papers (see Tables 1 and 3) reported on FOI associated with using modern contraceptive methods, one paper each discussed FOI and cervical screening [5], uptake of human papillomavirus (HPV) vaccinations [25], use of human immunodeficiency virus (HIV) prevention products [17]; three further studies explored the link between FOI and abortion [6–8]. For ‘infertility consequences’ there were 16 studies (across 17 publications) and two review papers with relevant data (see Tables 2 and 3).

The following text summarises key study characteristics from Tables 1 to 3.

### Study design

The majority of studies (*n* = 19) described the methodology as solely qualitative descriptive [5, 7, 8, 13, 18–24, 26, 27, 31, 35–40] or qualitative combined with other methods (*n* = 11 studies) in mixed methods research projects [6, 10, 14–17, 29, 30, 32–34]. Other qualitative studies were anthropological (*n* = 2) [42, 43], ethnographical (*n* = 3) [28, 44, 45] and phenomenological (*n* = 1) [41] designs.

### Country of origin

**Fig. 2:**
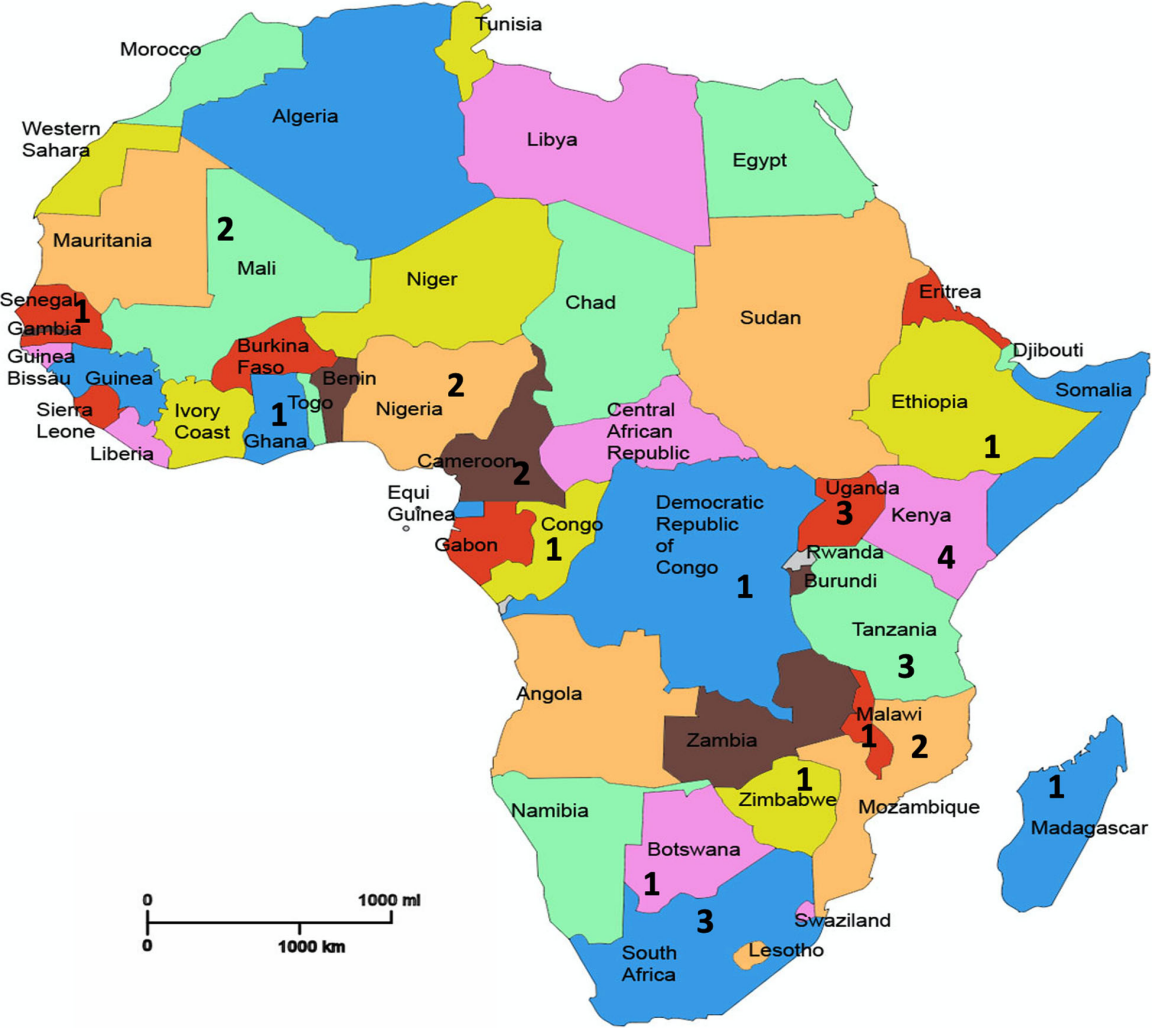
Number of studies identified per African country

The countries of origin primarily included Ghana (*n* = 8) [5, 13, 29, 31, 35, 38–40], Kenya (*n* = 4) [5, 26, 29, 41], Uganda (*n* = 3) [14, 19, 20], Tanzania (*n* = 3) [25, 28, 34], South Africa (*n* = 3) [24, 32, 36]; Cameroon (*n* = 2) [8, 43], Mozambique (*n* = 2) [15, 42], Mali (*n* = 2) [18, 33] and Nigeria [6, 7]. One research study was also conducted in each of the following countries Madagascar [27], Congo [22], Ethopia [16], Malawi [45], Rwanda [30], The Gambia [10], Zimbabwe [37] and Botswana [44]; and one across Malawi, South Africa, Uganda and Zimbabwe [17]. See Fig. 2 for number of studies across African countries.

### Participants

In the ‘triggering infertility’ studies, the participants included female [5, 13, 16, 20, 22, 23, 29] and male [5, 13, 16, 20, 22, 23, 25, 29] participants, adolescent boys [15, 18, 19, 29] and girls [15, 18, 19, 29]. Participants were described as: being married [16], married with children [22, 23], and women that were sexually active [7, 17, 26] or not [7], with experience of pregnancy [14, 21, 22, 24, 28] having had an abortion [8], fertility problems [6], having had or not cervical screening [5], or students attending local schools [25, 27]. Nine studies additionally reported views of traditional healthcare professionals [23, 25, 27], health workers [23, 25, 27–29] community leaders [13, 28, 29], religious leaders [25, 27, 29], peer educators [18], family planning service providers [20], aid workers [27], policy workers [20], teachers [25, 29], parents [25].

Participants in the ‘infertility consequences’ studies were couples (*n* = 3) [30, 38, 39], women (*n* = 9) [10, 31–35, 41–43], men (*n* = 2) [40, 45] or combination thereof (n = 3) [36, 37, 44] Also represented were traditional healthcare professionals or healers [38, 39, 42], religious leaders [38, 39] and managers of insurance schemes [38, 39]. Participants were described as having fertility problems (diagnosed or not, in treatment or not) (*n* = 12) [10, 30–34, 37, 40–44] seeking treatment in gynaecological and obstetric clinics (*n* = 1) [35] or being childless (*n* = 3) [38, 39, 45]. Three studies also explored the perspectives of fertile women [34, 42, 44].

### Types of questions from which FOI data emerged

Among ‘triggering infertility’ studies FOI data was reported to emerge from questions about family planning [13, 15, 21, 24, 27, 28] opinions thereof [19, 24, 27], barriers to use [18, 22, 26, 28, 29], knowledge of family planning [26, 28], side effects [29] and sources of influences [28]. Two studies asked specific questions about FOI [18, 29]. Aside from family planning, one study each explored the reasons for not wanting cervical cancer screening [5], HPV vaccination and its barriers [25] and abortion [14].

In ‘infertility consequences’ FOI data was reported to emerge from questions about infertility in the following domains: social (*n* = 7) [10, 30, 32, 34, 35, 40, 41], emotional/psychological (*n* = 5) [10, 30, 37, 40], economic (*n* = 1) [30], cultural and belief systems (*n* = 4) [30, 34, 37, 41] or personal experiences (*n* = 7) [10, 33–36, 41, 44]. Only one study asked questions about feared consequences specifically [44].

Questions were not provided for one study on HIV prevention [17], five publications on infertility consequences [31, 38, 39, 42, 45] and two studies exploring both topics [6–8]).

## Mapping of research findings about fears

### Fears in ‘triggering infertility’ studies

A fear presented across all included studies was that infertility could be triggered by using modern family planning methods [3, 4, 7, 8, 13–16, 18–21, 23, 24, 26–29, 46, 47]. This fear was expressed as permanent sterility or infertility [4, 6–8, 18, 22, 24, 26, 28, 46], temporary or delayed fertility [14, 20, 26, 28] or fertility not returning once contraception was stopped [14, 16]. These reports were in relation to hormonal methods (oral and injectable) [4, 6, 7, 14, 18–20, 23, 24, 26] and long acting and permanent contraceptive methods such as intrauterine devices or implant [3, 6, 14–16, 23, 24]. Only one review paper [47] cited a study that reported a link between FOI and condom use.

Fear of infertility was also reported in relation to abortion; women feared that unsafe methods could leave women infertile [6–8] and some condemned the use of induced abortion because of FOI [14]. Future infertility was also cited as a possible consequence of cervical cancer screening uptake [5], HPV vaccination of primary school girls in Tanzania [25] and use of the vaginal ring as an HIV prevention product [17].

### Explanations for fears in ‘triggering infertility’ studies

Fourteen studies and three of the review papers provided detailed descriptions of why reproductive or health choices were perceived to affect fertility (see Additional file 4 Explanations).

#### Accumulation and blockage

Women believed that oral contraceptive pills stayed in the womb and accumulated [18, 28]; men believed they spread throughout the body [28] or blocked up the reproductive organs [18]. A perceived consequence of hormonal contraceptives was too much or too little bleeding which was seen as affecting fertility [18]. Self-injection were associated with excessive bleeding accumulating in the womb [19, 23]. Pills and self-injections were perceived to prevent pregnancy through blocked blood [15, 46] or a blocked uterus [18, 28, 29].

#### Structural damage

There was a belief that ovarian damage could be caused by the HPV vaccine [25], contraceptive self-injection [19], intrauterine device (IUD) [14] or family planning in general [28]. Family members thought that the HPV vaccine acted to “disorder and destroy the eggs” [25]^.pg.5635^. Women and religious leaders used terms such as “burns eggs” [28].^pg.6^, “wasted eggs” or “kills God’s eggs” [28]^p.8^ for effects of hormonal contraceptives. [Repeated] abortions at a young age [8] or using hormonal contraceptives were thought to damage or spoil the womb [29, 46]. Women, men and healthcare providers believed modern contraceptive methods affected fertility by causing the womb to become “weak” [29]^.pg.350^, “thin [29]^.pg.351^” or “tired” [46]^.pg.10^. Women with fertility problems thought that having an abortion would spoil or destroy the womb [6]. Adolescent peer educators believed that the oral contraceptive pill worked by stopping implantation rather than ovulation [18]. Men, women and religious leaders reported that hormonal contraceptives killed [18, 28] or neutralized sperm [18].

#### Internal movement of contraceptive material

Pregnant women feared that the IUD would cause damage to nearby organs [14] or may go missing [13] which would result in the need for an operation that could affect future fertility. Men thought the IUD resulted in internal complications for young girls because their uterus was not developed [29]. Others thought the IUD would pass through the vagina into the womb [23] or that condoms would remain inside the body [46] therefore leaving women infertile. Women thought that the internal use of the speculum for cervical screening would cause infertility but did not elaborate on the specific mechanism [5].

#### Deliberate toxicity and contamination

There was a widespread belief among participants of a study on the vaginal ring for HIV prevention that the drugs inside the ring had been put there to deliberately cause infertility “to limit the Black population” [17]^, p6^. Men and women said that oral contraceptive pills or self-injections entered the blood stream and intentionally contaminated the blood [7, 18] or infiltrated blood to “kill all the germs that cause ovulation”^pg. 193^ [32].

#### Behavioral effects impeding sexual activity

Women experiencing vaginal dryness when using hormonal contraceptives reported it caused a loss of libido contributing to their inability to achieve pregnancy [20]. Others believed that the husband could be harmed during sex if the women used an IUD, also leading to childlessness [3].

### Fears in ‘infertility consequences’ studies

Sixteen studies (across 17 publications) and three review papers reported on the feared consequences of infertility (see Additional file 5 Consequences).

#### Fears of marital / partnership disruption

Men feared disapproval from their families and women feared partners would leave them when couples remained childless after cohabitation [36]. Married women feared the marriage would end in divorce [9, 41–43] or infidelity if they did not become pregnant [43]. Infertile men who had previously divorced feared that on becoming married again the next wife would leave them too because of their infertility [45]. Women that were not yet pregnant voiced fear that the husband would take another wife [9, 33] because relatives were pressurizing the husband [34]. Similar fears were expressed by childless women having perpetual fear of rivals (co-wives) [38, 39] and of tension between wives [10].

#### Fears of lower social standing

Wives expressed fear that their husband would listen to relatives and send her away [34] and feared mistreatment by their mother in law [38, 39]. Other childless women feared being isolated and left alone in their life [33, 37] or feared not having any true friends [41]. Men who were childless feared being openly insulted and disgraced [38, 39] or laughed at [36]. Women feared that an infertility status would label them with derogatory terms for being barren, i.e., “moopa” [44]^.pg.97^. Concerns about their future social status led women to fear being “condemned” [4]^.pg.111^.

#### Fear of future economic hardships

Infertile women and men voiced fear of economic difficulties in old age as they would lack the support of children [32] which was considered a daunting prospect [30, 31]. Participants feared losing properties and becoming impoverished [38, 39]; as well as losing financial support [37]. This included their property being taken by others after their death [30] and fear about the day of their funeral in which children play an important role [30].

#### Other fears

Men reported unspecified emotions related to fear (e.g., worries/sadness and fear) [40]. Men’s fears of sterility over-shadowed fears of HIV/AIDS (Acquired Immune Deficiency Syndrome) [44]. Women feared that witchcraft prevented the doctors from finding a cause for childlessness [32].

### Reported consequences of FOI

Fear of infertility was reported to cause a slow uptake of family planning [28], a switch to different or less effective family planning among adolescents and young women with no history of any fertility problems who had never been pregnant [15, 18], for participants to rely on abortion instead of contraception [7], to incorrectly use the vaginal ring [17], to abstain from using family planning methods [22] (e.g., hormonal contraceptives [18, 23, 26, 29], implants [26], injectables [19]), the HPV vaccination [25] and cervical cancer screening [5], or to discontinue use of injectables [19, 20, 26] and hormonal contraceptives [23]. FOI was reported to cause women to either not use family planning or use it incorrectly to prove their fertility or avoid infertility [6–8].

Regarding attitudes, a belief in the community and community leaders was that due to FOI young women (especially the nulliparous) should not start contraception [14, 20, 22, 28] or that injectables should only be recommended for women who already had children [26]. Wives reported that husbands/partners disapproved using family planning because of FOI [13, 21] consequently women sometimes used oral contraceptives without informing the husband [22]. Due to the possibility of being seen as at risk for infertility from using contraceptives some women were fearful of going to health centres for family planning [29].

Due to FOI and possible permanent childlessness the behaviour of not-yet fertile and infertile was affected. Among cohabitating couples, men broke promises of marriage if the woman had not produced a child during cohabitation [36]. Sometimes husbands of infertile women took second wives [30, 36, 37] or were encouraged by family members to abandon childless wives [30]. In the case of male infertility women reported that they would get pregnant through extra marital sex [10, 30, 37, 39] but that they kept it a secret from their husbands [30, 36], though some reported not doing this in case the husband knew of his infertility [36]. Traditional healers and spiritual leaders were consulted when pregnancy was not achieved [32, 33, 36, 39, 41, 45]. Traditional intervention could involve herbs [32, 33, 38], rituals [33, 39], sacrifices [33, 39], casting out of ancestral spirits [36], sexual preparations and remedies [37], therapeutic sex with healers [37] and other traditional fertility enhancement procedures [37]. Fear of infertility was also associated with religious practices (e.g., prayer, fasting) or divine interventions [30, 33, 39, 41, 45]. Men and women sought biomedical treatment [18, 47, 49, 50] but some kept treatment secret [30, 37].

Other behavioural consequences for childless or infertile women were relying on alcohol [37, 38]. Some childless couples adopted the children conceived in polygamous relationships [37], looked after the children of others [30, 37], fostered [30] or re-engaged with other goals (e.g., economic) [38].

### Country differences

There were too few studies per country to carry out and in-depth comparison of fears between countries. Available data do not appear to show systematic differences (see Additional files 4 and 5).

## Conclusion

Fear of infertility is a phenomenon that should be studied in its own right. Evidence for FOI was reported in many sub-Saharan African countries and expressed by a wide range of people (e.g., men, women, young people, teachers, healthcare professionals, religious leaders, and the childless). Two types of fears were identified in included qualitative studies: fear that specific health or reproductive choices (e.g., family planning) would trigger future infertility and fear of dire consequences of infertility for oneself. Many explanations were offered for why choices could affect fertility, and many feared consequences described. Fear of infertility was reported to affect behaviour in important ways but was rarely the main topic of the included studies. Rigorous prospective studies are needed to understand origins of FOI, optimise health messaging about FOI and minimise its consequences on health behaviour and outcomes. Integrating fertility in sexual and reproductive health policies could stimulate necessary partnerships where FOI was observed (e.g., family planning, HPV vaccination, HIV prevention, infertile communities) and support de-stigmatisation of infertility, an important precursor of FOI in the community.

Fears were reported to impact behaviour, for example abstaining altogether from using family planning, switching from more to less effective contraception and missing opportunities for prevention (screening, vaccination). Additionally, people fearing the consequences of permanent sterility engaged in health-behaviours that would not resolve fertility problems including some that might have caused or exacerbated fertility problems (e.g., unprotected sex [37]). Despite these reported effects not much importance seems to be placed on FOI in existing research. FOI was the focal study topic in only 5% of included studies. Even if FOI affected a small proportion, its impact could be significant given suggested effects on behaviour. Estimating prevalence of FOI and determining its impact on behaviour in rigorously designed prospective studies is warranted.

Fears are *constructed* expectations of negative outcomes [1]. As such FOI could originate in genuine threats (e.g., genuine severe consequences of infertility, unsafe abortion), distorted or poorly understood facts (e.g., delay in return to fertility after injectables) or motivated spread of misinformation (e.g., leaders exhorting malevolent motives of white researchers [2, 17]). How ever constructed, FOI should be explicitly addressed in health education with men and women of all ages making health and reproductive choices [51]. Providers of education (e.g., teachers, community leaders) also reported fears and possibly are transmitters of FOI so they too could benefit from more training about links between fertility and reproductive or health choices (e.g., family planning, screening). It will be more difficult to tackle fear of the dire consequences of infertility as this is likely to require wider societal change to de-stigmatise infertility and childlessness. Although we dealt with the two types of fears separately, we believe these to be causally related. People making choices would fear future infertility less if infertility caused less dire consequences for those affected. Current initiatives to increase understanding and awareness of causes of fertility problems [49], integration of fertility care in sexual and reproductive health policy [52] and inclusion of fertility topics in national education curriculums should help. We agree with recent calls for integration at such levels [50] because it would stimulate the necessary partnerships across areas where FOI was observed and strengthen potential for timely research and health education. Future research could also benefit from cross country comparisons to ensure that local beliefs are adequately considered and addressed.

### Limitations

We believe we have mapped the main concepts and topics to emerge from research referring to FOI. However, the search strategy for ‘fear’ is complicated by the many ways such fears could be expressed (e.g., worry, concern, threat, afraid) and the fact that FOI is not a MeSH (Medical Subject Headings) term. Consequently, the literature on FOI could be much larger (though not necessarily more informative). We used a rapid review scoping method which entails the usual methodological limitations of this approach (e.g., limited search, lack of quality assessment, not all reproductive choices). For example the paper would be excluded if it did not identify fear related to current or future infertility in the abstract, or as a succinct theme heading. This means that some studies that could have indirectly related to effects of fear on infertility could be omitted. We selected only qualitative studies and in so doing we missed the gains that could have been achieved with quantitative data (e.g., proportion of specific populations reporting FOI). We provided a simple thematic account of FOI, but a more in-depth analysis could have provided useful elaboration. For example, we did not pay attention to the development, maintenance, sharing or resolution of FOI but this would be worth investigating in future research [53]. Finally, the two fears seem to occur in different populations, moments in the life span and readiness to achieve pregnancy/father a child. Future reviewers and researchers may choose to deal with one or both fears, but we suggest that causal relations between these should not be ignored.

In conclusion, fear of infertility concerns fear of triggering infertility and fear of the dire consequences of infertility to oneself. Fear of infertility should be addressed and its potential impact on reproductive and health choices the subject of further investigation.

## Supplementary information

**Additional file 1.** Search History_Medline.docx Illustrative Search Strategy

**Additional file 2.** PRISMA-ScR Checklist.docx PRISMA checklist for scoping reviews

**Additional file 3.** excluded studies.docx Studies excluded (DOCX 27 kb)

**Additional file 4.** Explanations.docx Explanations for why choices affected fertility

**Additional file 5.** Consequences.docx Feared consequences of infertility

## Data Availability

All data generated or analysed during this study are included in this published article [and its supplementary information files].

## Abbreviations

AIDS: Acquired immunodeficiency disorder
FOI: Fear of Infertility
HIV: Human immunodeficiency virusIUD = intrauterine device
HPV: Human papillomavirus
MeSH: Medical Subject Headings
PRISMA: Preferred Reporting Items for Systematic Reviews and Meta-Analyses
PRISMA_SCr: Preferred Reporting Items for Systematic Reviews and Meta-Analyses Extension for Scoping Reviews
