# Supplementary material for: A rapid scoping review of fear of infertility in Africa
Source: Reprod Health. 2020 Sep 14;17:142. doi: 10.1186/s12978-020-00973-0 (PMC7488744; doi:10.1186/s12978-020-00973-0)
Supplement: Supplementary file 1 — Additional file 1. Search History_Medline.docx Illustrative Search Strategy [file 12978_2020_973_MOESM1_ESM.docx]

Database: Ovid MEDLINE(R) ALL <1946 to January 28, 2019>

Search Strategy:

--------------------------------------------------------------------------------

1 fear.tw. (57314)

2 exp *Fear/ (16319)

3 1 or 2 (61995)

4 infertili*.tw. (46770)

5 childless*.tw. (1731)

6 sterility.tw. (14650)

7 infecundity.tw. (52)

8 subfecundity.tw. (125)

9 exp *Infertility, Female/ or exp *Infertility/ (45122)

10 4 or 5 or 6 or 7 or 8 or 9 (82254)

11 exp *South Africa/ or exp *Africa, Northern/ or Africa, Southern/ or exp *Africa, Eastern/ or exp *Africa, Central/ or exp *"Africa South of the Sahara"/ or exp *Africa/ or exp *Africa, Western/ (2273)

12 africa*.tw. (207606)

13 exp *Developing Countries/ (26574)

14 "developing countr*".tw. (54635)

15 (poor resource* and (setting* or area* or countr* or region*)).tw. (197)

16 (low resource* and (setting* or area* or countr* or region*)).tw. (4402)

17 (africa* or Algeria or Angola or Benin or Botswana or Burkina Faso or Burundi or Cabo Verde or Cameroon or Chad or Comoros or Congo or Cote d'Ivoire).tw. (230200)

18 (Djibouti or Egypt or Equatorial Guinea or Eritrea or Eswatini or Swaziland or Ethiopia or Gabon or Gambia or Ghana or Guinea or Guinea-Bissau).tw. (142096)

19 (kenya or Lesotho or Liberia or Libya or Madagascar or Malawi or Mali or Mauritania or Mauritius or Morocco or Mozambique or Namibia or Niger or Nigeria or Rwanda).tw. (78134)

20 ((Sao Tome and Principe) or Senegal or Seychelles or Sierra Leone or Somalia or Sudan or Tanzania or Togo or Tunisia or Uganda or Zambia or Zimbabwe).tw. (53265)

21 11 or 12 or 13 or 14 or 15 or 16 or 17 or 18 or 19 or 20 (515727)

22 3 and 10 and 21 (43)

23 limit 22 to english language (42)
