# Supplementary material for: A rapid scoping review of fear of infertility in Africa
Source: Reprod Health. 2020 Sep 14;17:142. doi: 10.1186/s12978-020-00973-0 (PMC7488744; doi:10.1186/s12978-020-00973-0)
Supplement: Supplementary file 3 — Additional file 3. excluded studies.docx Studies excluded (DOCX 27 kb) [file 12978_2020_973_MOESM3_ESM.docx]

**Additional material 1**

**Studies excluded on full text screening**

1. Folkvord et al 2005^1^: Male infertility in Zimbabwe

*Reason for exclusion: No mention of fears related to current or future infertility*

1. Moyo 2013^2^: Indigenous knowledge systems and attitudes towards male infertility in Mhondoro-Ngezi, Zimbabwe

*Reason for exclusion: No mention of fears related to current or future infertility*

1. Capurchande et al 2017^3^: "If I have only two children and they die… who will take care of me?" - A qualitative study exploring knowledge, attitudes and practices about family planning among Mozambican female and male adults

*Reason for exclusion: No mention of fears related to current or future infertility*

1. Chipeta et al 2010^4^: Contraceptive knowledge, beliefs and attitudes in rural Malawi: misinformation, misbeliefs and misperceptions

*Reason for exclusion: No mention of fears related to current or future infertility*

5. Nachinab et al 2018^5^: Child adoption as a management alternative for infertility: A qualitative study in rural Northern Ghana

*Reason for exclusion*: Adoption

6. Nwobodo and Isah 2011^6^: Knowledge, attitude and practice of child adoption among infertile female patients in Sokoto north-west Nigeria

*Reason for exclusion* : Adoption

7. Oladokun et al 2009^7^: Acceptability of child adoption as management option for infertility in Nigeria: Evidence from focus group discussions

*Reason for exclusion* :Adoption

8. Stuckenbruck and Roby 2017^8^: Navigating uncharted terrain: Domestic adoptions in Kenya

*Reason for exclusion* Adoption

9. Castle 1990^9^: Observations on abortion in Zambia

*Reason for exclusion: No mention of fears related to current or future infertility*

10. Coeytaux et al 1998^10^: Induced abortion in sub-Saharan Africa: what we do and do not know

*Reason for exclusion: No mention of fears related to current or future infertility*

11. Hollos 2003^11^: Profiles of infertility in southern Nigeria: women's voices from Amakiri

*Reason for exclusion: No mention of fears related to current or future infertility*

12. Hollos et al 2014^12^: Women in limbo: Life course consequences of infertility in a Nigerian community

*Reason for exclusion: No mention of fears related to current or future infertility*

13. Ibisomi et al 2014^13^: Childlessness in Nigeria: Perceptions and acceptability

*Reason for exclusion: No mention of fears related to current or future infertility*

14. Moyo and Muhwati 2013^14^: Socio-cultural perspectives on causes and intervention strategies of male infertility: A case study of Mhondoro-Ngezi, Zimbabwe

*Reason for exclusion: No mention of fears related to current or future infertility*

15. Nguimfack et al 2016^15^: Brief report: A Cameroonian woman's cultural-bound experience of infertility

*Reason for exclusion: No mention of fears related to current or future infertility*

16. Nieuwenhuis et al 2009^16^: The impact of infertility on infertile men and women in Ibadan, Oyo State, Nigeria: a qualitative study

*Reason for exclusion: No mention of fears related to current or future infertility*

17. Pedro and Faroa 2017^17^: Exploring the lived experiences of infertility treatment and care by involuntarily childless women

*Reason for exclusion: No mention of fears related to current or future infertility*

18. Richards 2002^18^: "Spoiling the womb": definitions, aetiologies and responses to infertility in north west province, Cameroon

*Reason for exclusion:* No mention of fears related to current or future infertility

19. Stanback and Twum-Baah 2001^19^: Why do family planning providers restrict access to services? An examination in Ghana

*Reason for exclusion: No mention of fears related to current or future infertility*

20. Yebei 2000^20^: Unmet needs, beliefs and treatment-seeking for infertility among migrant Ghanaian women in the Netherlands

*Reason for exclusion: No mention of fears related to current or future infertility*
