# Supplementary material for: A rapid scoping review of fear of infertility in Africa
Source: Reprod Health. 2020 Sep 14;17:142. doi: 10.1186/s12978-020-00973-0 (PMC7488744; doi:10.1186/s12978-020-00973-0)
Supplement: Supplementary file 4 — Additional file 4. Explanations.docx Explanations for why choices affected fertility [file 12978_2020_973_MOESM4_ESM.docx]

**Supplementary file 4: Nature of fears in included studies describing fear of triggering infertility (where provided)**

| **Author** | **Age (years)** | **Country** | **Mechanism** |
| --- | --- | --- | --- |
| 1. Castle 2003[18] | 15 to 19 | Mali | [Hormonal contraceptives] little (or too much) bleeding can affect fertility  [Oral contraceptive pills] block up reproductive organs, accumulate in the uterus, stops the egg from implanting in the womb  [Contraceptive self-injections] block the uterus or kill or neutralize sperm, enters blood stream and prevents pregnancy |
| 2.Cover et al 2017[19] | 15 to 19 | Uganda | [Contraceptive self-injection] blood accumulating in the uterus and harm to ovaries |
| 3.Hytell et al 2012[20] | Mixed 18 to 60 | Uganda | [Hormonal contraceptives] vaginal dryness and loss of libido led to lessening sexual desire |
| 4.Morse et al 2012[14] | < 20 to > 30 | Uganda | Internal nature of the intrauterine device and fears of damage to nearby organs causing a need for surgery.  [Oral contraceptive pills] damage the eggs and leads to infertility |
| 5.Capurchande et al 2016[15] | 15 to 24 | Mozambique | [Modern contraceptive methods] blood blockage |
| 6.Adongo et al 2014[23] | Not reported | Ghana | [Contraceptive self-injection] blood accumulates in your womb  [IUD] passes through vagina and shifts to the womb |
| 7.Chebet et al 2015[28] | 18 to 43 | Tanzania | [Oral contraceptive pills] blocked uterus due to pill accumulation; pills spread through the body  [FP] wasted or kills sperm or eggs, burns eggs |
| 8.Sedlander et al 2018[29] | 13 to 65 | Kenya | [Modern contraceptive methods} blocked uterus, damage to the uterus, spoiling reproductive system; make womb “weak” or “thin, complications for young girls due to fact reproductive system not yet developed |
| 9.Koster 2010[6] | 15 to 49 | Nigeria | [Hormonal contraceptives and IUD] ‘spoiling’ or ‘destroying’ the womb |
| 10.Otoide et al 2001[7] | 15 to 24 | Nigeria | [Oral contraceptive pills] entered blood stream and directly contaminated the blood;  [IUD] going “missing” and needing operation for removal |
| 11.Schuster et al 2005[8] | not reported | Cameroon | [Abortion] repeated abortion at a young age |
| 12.Lunsford et al 2017[5] | 25 to 49 | Kenya | [Cervical screening] speculum causes infertility |
| 13.Remes et al 2012[25] | 19 to 55 | Tanzania | [HPV vaccination] disorder and destroy the eggs |
| 14.Chitukuta et al 2019[17] | 14 to 42 | Zimbabwe | [HIV prevention product] toxicity; vaginal administration of ring used to deliberately cause infertility |
| 15. Polis et al 2018[46] | [review] | 11% of studies from Africa | [Contraception induced bleeding] Blocked blood (amenorrhea) could cause the womb to “get tired” or that excessive bleeding would lead to infertility |
| 16.Ackerson and Zielinski 2017[47] | [review] | Sub-Saharan Africa | [Traditional contraceptive methods] condoms could remain inside the woman |
| 17.Daniele et al 2017[3] | [review] | Low- and middle-income countries  Including Africa | [IUD] harm to the husband during sex |

Key: FP: Family planning; HPV: Human papilloma virus; IUD: Intra uterine device
