# Supplementary material for: A rapid scoping review of fear of infertility in Africa
Source: Reprod Health. 2020 Sep 14;17:142. doi: 10.1186/s12978-020-00973-0 (PMC7488744; doi:10.1186/s12978-020-00973-0)
Supplement: Supplementary file 5 — Additional file 5. Consequences.docx Feared consequences of infertility [file 12978_2020_973_MOESM5_ESM.docx]

Table of perceived (or actual) fears about consequences of infertility

| **Author** | **Country** | **Perceived (or actual) fears** |
| --- | --- | --- |
| 1.Dhont et al 2011[31] | Rwanda | Fear of old age without support of child(ren), fear of property taken by others after their death, fear about the day of their funeral in which children play an important role & fear of not being able to carry on the family line (women concerned about the man’s family) |
| 2.Dierickx et al 2018[10] | Gambia | Fear of divorce; fear of polygamy & fear of tension between fertile and infertile co-wives |
| 3.Donkor et al 2017[32] | Ghana | Fear of becoming old without carrying children & fear of losing husband (to polygamy) |
| 4.Dyer et al 2002[33] | South Africa | Fear that witchcraft preventing the doctors from finding a cause for childlessness |
| 5.Fledderjohann 2012[36] | Ghana | Fear of infidelity and divorce |
| 6.Gerrits et al 1997[43] | Mozambique | Fear that family would die out; Fear of divorce |
| 7.Hess 2018[34] | Mali | Fear of being alone & fear of husband taking another wife |
| 8.Hollos and Larsen 2008[35] | Tanzania | Fear that relatives will pressure husband to marry another woman & fears that her husband will listen to relatives and send her away |
| 9.Kamau 2012[42] | Kenya | Fear of being divorced, fear of not having true friends & fear husband will leave the wife |
| 10.Mabasa 2005[37] | South Africa | In cohabitating couples, fear of disapproval from extended families, fear that people would laugh at [fatherless, possibly infertile] him & women fear that partners would leave; not telling people about infertile state. |
| 11.Runganga et al 2001[38] | Zimbabwe | Fear of isolation & fear of losing financial support |
| 12.Tabong and Adongo 2013a/b[39,40] | Ghana | Men who are childless fear being openly insulted and disgraced, fear of mistreatment by mother in law, perpetual fear of husband acquiring rivals (new wives) & fear of losing properties and becoming impoverished |
| 13.Upton and Dolan 2011[45] | Botswana | Fears of sterility overshadow fears of HIV/AIDS [have risky sex to be fertile] & fears of infertility status (being labelled as a “moopa” pg 97 |
| 14.Naab and Kwashie 2018[41] | Ghana | Worries/sadness and fear (not specified) were reported by the men |
| 15.Feldman-Savelsberg 1994[44] | Cameroon | Fear of infertility reflects women’s feelings of vulnerability within these broader contexts (culinary analogy) |
| 16. Parrott 2014[46] | Malawi | In men, fears that on becoming married again that the wife will leave because of the husband’s infertility |
| 17.Dyer and Patel 2012[49] | Developing countries  Africa (n=13) | Infertile women, and at times men, voiced fear of economic difficulties in old age as they would lack the support of children |
| 18.Van Balen and Bos 2009[9] | Poor resource areas  Sub-Sahara Africa (n=19) | Fear of the husband taking a second wife or divorcing the childless woman |
| 19.Williamson et al 2009[4] | Developing countries  Sub-Sahara Africa (n=6) | Future social status was highly dependent on future fertility, concerns about which led to a fear of being "condemned" (page 111). This was reinforced by the young women's partners, their parents and wider society |

Note. Reference citation follows author name in square brackets.
